# Digital Image Analysis-Based Evaluation of Claudin-1 and Claudin-7 Delocalization in Cutaneous Squamous Cell Carcinoma and in Its Precancerous State

**DOI:** 10.1155/2022/2750193

**Published:** 2022-04-06

**Authors:** Lina Xu, Yunlei Pan, Shunli Tang, Juan Bai, Yinhua Wu, Jianjun Qiao, Hong Fang

**Affiliations:** Department of Dermatology, The First Affiliated Hospital, College of Medicine, Zhejiang University, Hangzhou 310003, China

## Abstract

Accumulating evidence has revealed that delocalization of the transmembrane proteins, Claudin-1 and Claudin-7, to the cytoplasm and/or nucleus occurs in various tumors. However, their subcellular distribution in terms of the membrane, cytoplasm, and nucleus and relationship with signaling pathways have not been elucidated during carcinogenesis. We first determined the expression of these proteins in the membrane, cytoplasm, and nucleus using ImageJ software and automatically collected the immunohistochemical quantification of dysplasia (actinic keratosis (AK)), carcinoma in situ (CIS; Bowen's disease (BD)), and invasive cutaneous squamous cell carcinoma (SCC) for digital image analysis (DIA). The activity of p-ERK, p-AKT, and p-mTOR and their correlation with subcellular Claudin-1 and Claudin-7 were also performed. Finally, we validated Claudin-1 and Claudin-7 delocalization at the cytoplasm and nucleus in cultured human normal keratinocytes and cutaneous SCC cells. Claudin-1 and Claudin-7 were delocalized as revealed by membranous, cytoplasmic, and nuclear staining in sun-exposed skin, AK, BD, and SCC. In BD, both membranous and cytoplasmic Claudin-1 (nuclear Claudin-1 decrease but no significant difference) were higher than AK, while Claudin-7 almost had the opposite situation. In SCC, cytoplasmic and nuclear Claudin-1 (membranous Claudin-1 no significant difference) was lower than in AK and sun-exposed skin, while Claudin-7 had higher membranous and cytoplasmic but lower nuclear expression. Moreover, p-AKT and p-mTOR (but not p-ERK) were downregulated in the SCC. Subcellular Claudin-1 and Claudin-7 were not only correlated with each other, but also correlated with p-ERK in BD and p-AKT and p-mTOR in SCC. Together, these results imply the delocalization of Claudin-1 and Claudin-7 and their correlation with MAPK/ERK and PI3K-AKT-mTOR signaling pathways in tumorigenesis and infiltration in cutaneous SCC.

## 1. Introduction

The incidence of cutaneous squamous cell carcinoma (SCC) is on the rise, making it the second-most-common keratinocyte-derived carcinoma [1]. Its classic multistep carcinogenesis involves several events, ranging from cumulative sun exposure to precursor actinic keratosis (AK), carcinoma in situ (CIS; Bowen's disease (BD)), and invasive cutaneous SCC. There are at least two pathways involved in cutaneous SCC: phosphatidylinositol 3-kinase- (PI3K-) AKT-mammalian target of rapamycin (mTOR) pathway and the mitogen-activated protein kinases/extracellular signal-regulated kinase (MAPK/ERK) pathway. Overactivation of PI3K-AKT-mTOR is observed in cutaneous SCC and its inhibition suppresses the growth of human cutaneous SCC, both *in vitro* and *in vivo* [2–5]. The level of phosphorylated-ERK1/2 (p-Erk1/2) is correlated with the size of cutaneous SCC, but not with its metastasis [3]. However, the dynamic activities of these pathways in tumorigenesis and infiltration of cutaneous SCC are not well understood and, therefore, need to be clarified.

Claudins contain 27 transmembrane-protein-family members which play important roles in tight junction (TJ) formation and regulate paracellular permeability, as well as cell polarity in epithelial and endothelial cells. The TJ integrity is often decreased in cancerous cells due to dysregulation of tight junction proteins (TJPs), such as up- or downregulation or delocalization of Claudins. Claudin-1 expression in the membrane, cytoplasm, and nucleus is altered in human malignancies including colon cancer [6], nasopharynx carcinoma [7], thyroid carcinoma [8], breast cancer [9], and melanoma [10, 11]. Moreover, subcellular Claudin-1 correlates with aggressive tumor behaviors such as enhanced cell migration, invasion, and proliferation, as well as decreased apoptosis [7, 8]. Claudin alterations have also been reported in cutaneous SCC and its precancerous lesions [12, 13], whereby a faint or strong cytoplasmic, rather than primary membranous, localization of Claudin-1 was found to be parallel with the cutaneous SCC carcinogenesis process. Additionally, Claudin-1 expression was found to be heterogeneous between keratinized and unkeratinized cutaneous SCC [14]. However, digital image analysis of membranous/cytoplasmic/nuclear Claudin-1 and Claudin-7 in multisteps of carcinogenesis in cutaneous SCC has not been performed. In this study, two auto-scored plugins of ImageJ software, ImmunoMembrane plugin exclusive for membrane proteins [15] and IHC profiler plugin for cytoplasmic/nuclear molecules [16], were developed for a series of cutaneous SCC, BD, AK, and sun-exposed skin to avoid subjective visual and qualitative estimation.

Claudin-1 and Claudin-7 delocalization was determined using membranous, cytoplasmic, and nuclear staining of sun-exposed normal skin, AK, BD, and SCC. Both membranous and delocalized cytoplasmic Claudin-1 was increased in BD, compared with AK, but delocalized nuclear Claudin-1 was decreased in SCC. Similarly, both membranous and cytoplasmic Claudin-7 staining was found to be elevated in SCC when compared to AK and BD, while its delocalized nucleus staining was reduced. Furthermore, the relationships between Claudin-1 and Claudin-7 subcellular localization and their correlation with p-ERK, p-AKT, and p-mTOR were examined to analyze their alterations in sun-exposed skin, AK, BD, and SCC.

## 2. Materials and Methods

### 2.1. Skin Samples

Sun-exposed skin (apparently normal skin, abbreviated as N) samples were obtained from patients with head-face orthopedics. According to the diagnosis by two pathologists, all AK, BD, and SCC tissue sections obtained from the head and face regions were selected and fixed with paraffin. In addition, SCC samples were obtained from skins without metastasis. There were no age and gender differences among the patients. Skin samples were obtained with the patient's written informed consent after pathological diagnosis.

### 2.2. Immunohistochemistry (IHC)

All 4 *μ*m thick sections were first deparaffinized and rehydrated, followed by antigen retrieval by heating in a microwave oven for 30 min. The specimens were then treated with 3% H_2_O_2_ for 10 min to block endogenous peroxidase activity. All slides were incubated with primary antibodies for 1 h at room temperature and then with secondary antibody (DAKO) for 30 min at room temperature to detect the antibody-antigen complex. Subsequently, the samples were stained with a chromogenic substrate, 3,3′-diaminobenzidine tetrahydrochloride (DAB substrate chromogen, DAKO), for different durations according to our preliminary tests. The primary antibodies used were the following: Anti-Claudin1 (1 : 200; ab15098, Abcam, UK), Anti-Claudin7 (1 : 500; ab27487, Abcam), Anti-p-ERK (1 : 400; 4376S, Cell Signaling Technology, USA), Anti-p-Akt (1 : 250; 66444-1-Ig, Proteintech, USA), and Anti-p-mTOR (1 : 100; 2976S, Cell Signaling Technology).

### 2.3. Image Acquisition

Images were captured in triplicate at ×40 magnification using a light microscope (Leica™ DM2500) with CCD (Leica DFC320) by manual exposure and white balance with identical parameters for each marker. Identical contrast and brightness conditions were applied for each antibody. All the images were saved in the TIFF format.

### 2.4. ImageJ Analysis

The ImmunoMembrane plugin running in ImageJ was used to establish membrane-binding proteins. Firstly, regions of interest (ROI) for each slide were visually demarcated by two pathologists. Secondly, digital image analysis (DIA) was automatically performed by defined custom cutoff values using the ImmunoMembrane (IM) score (0-20 points), depending on the membrane staining intensity and completeness, finally the *M* score for each case.

At the same time, the DIA of the cytoplasmic and nuclear expression was automatically scored using the IHC profiler plugin running in ImageJ. The ROIs were selected before the staining intensity was automatically assigned into a four-tier system (high positive, positive, low positive, and negative) for each image, all of which were in percentage form. All data was presented as the average of the triplicates for each case.

### 2.5. Cell Culture

Human cutaneous squamous carcinoma A431 cell line and human immortalized HaCaT keratinocytes (Institute of Biochemistry and Cell Biology, Shanghai, China) were cultured in Dulbecco's modified Eagle's medium (DMEM), supplemented with both 10% heat-inactivated Fetal Bovine Serum (FBS, Invitrogen) and 1% antibiotic-antimycotic (Invitrogen) at 37°C and 5% CO_2_ in a 95% humidified incubator.

### 2.6. Protein Extraction and Western Blot (WB) Analysis

Nuclear and cytoplasmic proteins were isolated by Nuclear and Cytoplasmic Protein Extraction Kit (Beyotime, Haimen, China) following the manufacturer's protocols. After quantification determined by the Enhanced BCA Protein Assay Kit (Beyotime, Haimen, China), the protein samples were separated by sodium dodecyl sulphate-polyacrylamide gel electrophoresis (SDS-PAGE). Upper stacking gels (5%, 90 V) and lower separating gels (12%, 120 V) were orderly run containing 30 *μ*g samples, which were later transferred to polyvinylidene fluoride (PVDF) microfiltration membrane by Trans-Blot® Turbo™ Transfer System (BIO-RAD, Hercules, CA, USA) under 200 mA at 1 min/kDa. After TBST containing 5% skimmed milk sealing nonspecific sites for 1 h at room temperature, the PVDF membrane was incubated overnight at 4°C with corresponding antibodies against Claudin-1, Claudin-7, and Histone (15 kDa, AF1684, Beyotime) and enhanced by chemiluminescence (ECL) substrate (BIO-RAD, Hercules, CA, USA) and then quantified by the ChemiDoc™ Touch Imaging System (BIO-RAD, Hercules, CA, USA).

### 2.7. Statistical Analyses

Results are presented as mean ± SEM. Statistical significance was calculated using the paired *t*-test. Multivariate analysis was performed by two-way ANOVA using GraphPad Prism 5 (GraphPad Software, La Jolla, CA, USA). Scatterplot matrices and heatmaps are displayed by pairwise Pearson correlation coefficients using JMP statistical software release 10.0 (JMP, a trademark of SAS Institute).

## 3. Results

### 3.1. Claudin-1 and Claudin-7 Displayed Altered Membrane Expression and Delocalized Subcellular Location in SCC

**Figure 1:**
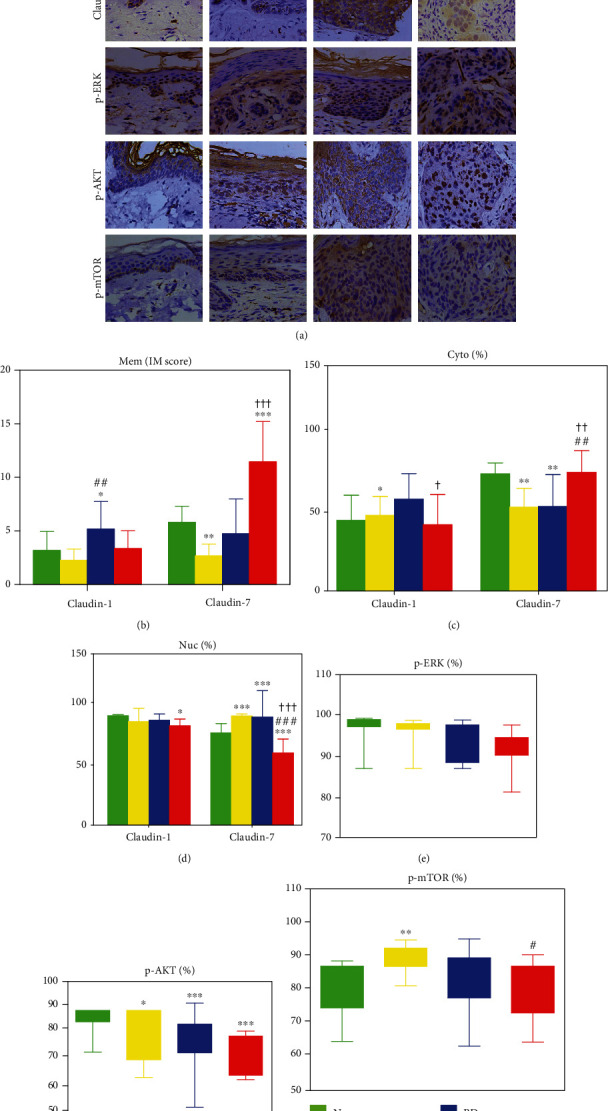
Immunohistochemical analysis. (a) Subcellular localization of Claudin-1 and Claudin-7 and p-ERK, p-AKT, and p-mTOR expression in sun-exposed skin, precancerous actinic keratosis (AK), in situ Bowen's disease (BD), and invasive cutaneous squamous cell carcinoma (SCC) (×400). Quantitative analysis of Claudin-1 and Claudin-7 immunostaining for (b) membrane (Mem, IM scores), (c) cytoplasm (Cyto, %), and (d) nucleus (Nuc, %) localization reflected by positive percentages in sun-exposed skin (green), AK (yellow), BD (blue), and SCC (red). A comparison of positive percentages of (e) p-ERK, (f) p-AKT, and (g) p-mTOR. Relative to sun-exposed skin: ^∗^*P* < 0.05; ^∗∗^*P* < 0.01; ^∗∗∗^*P* < 0.001. Relative to AK tissue: ^#^*P* < 0.05; ^##^*P* < 0.01; ^###^*P* < 0.001. Relative to BD tissue: ^†^*P* < 0.05; ^††^*P* < 0.01; ^†††^*P* < 0.001.

We first detected the expression of Claudin-1 and Claudin-7 in each tissue using IHC (Figure 1(a)) and subsequently analyzed their staining expression values by ImageJ (Table 1). Membranous Claudin-1 was upregulated in BD, relative to N and AK. Similarly, membranous Claudin-7 was significantly increased in SCC compared to BD, AK, and N (Figure 1(b)). The cytoplasmic Claudin-1 was increased in BD and N, but decreased in SCC compared to BD. Cytoplasmic Claudin-7 was obviously downregulated in AK and BD compared to N, but elevated in SCC compared to AK and BD (Figure 1(c)). Nuclear Claudin-1 was downregulated in SCC, while nuclear Claudin-7 was reduced in AK, BD, and SCC (Figure 1(d)).

### 3.2. p-AKT and p-mTOR (but Not p-ERK) Participate in Tumorigenesis and Infiltration in Cutaneous SCC

Intracellular staining revealed that the activation level of p-AKT was reduced (Figure 1(f)). p-mTOR increased in AK but decreased in SCC (Figure 1(g)) while the level of activated p-ERK was not altered (Figure 1(e)). Collectively, these findings suggest that the p-AKT and p-mTOR signaling pathways closely are associated with tumorigenesis and infiltration of cutaneous SCC.

### 3.3. Correlations among Subcellular Claudin-1 and Claudin-7 and p-ERK, p-AKT, and p-mTOR in Different Stages of Cutaneous SCC

**Figure 2:**
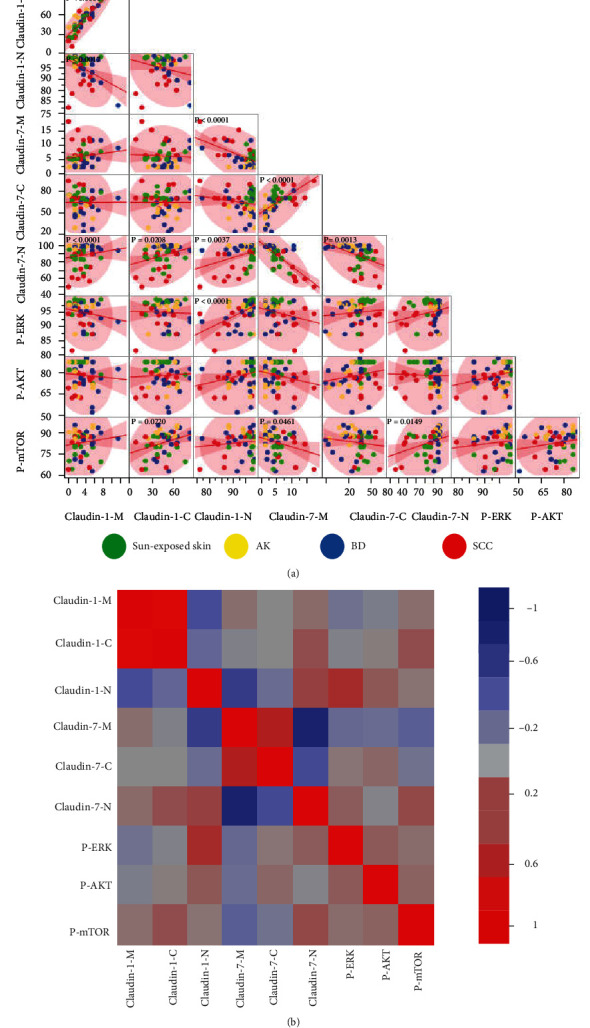
Data correlation analysis. (a) Scatterplot matrix indicating correlations within the entire data set (all four groups: sun-exposed skin, AK, BD, and SCC). Pairwise correlation analyses include the tight junction protein (TJPs) characteristics of membrane (Claudin-1-M, Claudin-7-M), cytoplasm (Claudin-1-C, Claudin-7-C), and nucleus (Claudin-1-N, Claudin-7-N) and signaling pathway proteins (p-ERK, p-AKT, and p-mTOR). Data about different groups is represented by different colors (sun-exposed skin, green; AK, yellow; BD, blue; SCC, red). The red line represents the pairwise linear regression slope, while the pink shaded clouds represent the 95% bivariate normal density ellipse. (b) Heatmap representation for the data set.

**Figure 3:**
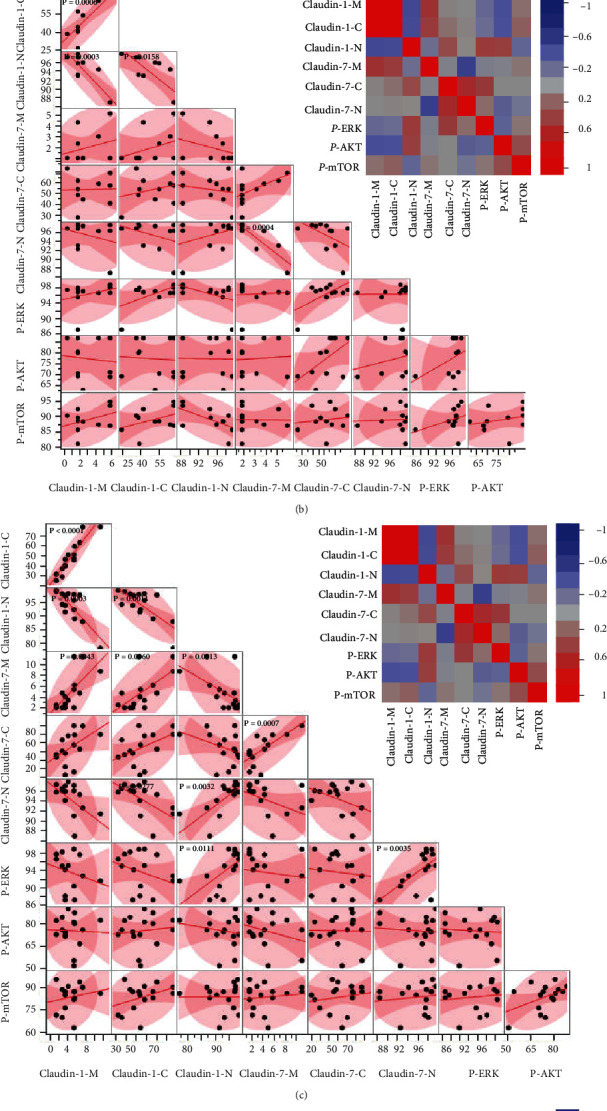
Data correlation analysis for different groups. The same correlation analysis as in Figure 2 for (a) sun-exposed skin, (b) AK, (c) BD, and (d) SCC.

Pairwise correlation analyses for TJPs (localization of membrane, M; cytoplasm, C; nucleus, N), p-ERK, p-AKT, and p-mTOR in all samples are shown in Figures 2 and 3. The data (Figures 2(a) and 2(b)) showed a moderate positive correlation between Claudin-1-N and p-ERK. p-mTOR exhibited a weak positive correlation with Claudin-1-C and Claudin-7-N, but a weak negative correlation with Claudin-7-M. Claudin-1-M had a strong positive correlation with Claudin-1-C, but a moderate negative correlation with Claudin-1-N, and a weak correlation with Claudin-1-C and Claudin-1-N. Although Claudin-7-M showed a moderate positive correlation with Claudin-7-C, it was not correlated with Claudin-7-N, and there was a weak negative correlation between Claudin-7-C and Claudin-7-N. Claudin-7-N exhibited a moderate negative correlation with Claudin-1-M, but a weaker positive correlation with Claudin-1-C and Claudin-1-N. Additionally, a moderate negative correlation was found between Claudin-7-M and Claudin-1-N.

For sun-exposed skin, a strong positive correlation was observed between Claudin-1-M and Claudin-1-C. Moreover, Claudin-7-N was positively correlated with Claudin-7-M, but negatively correlated with Claudin-7-C. There was no correlation between subcellular TJPs and p-ERK, p-AKT, or p-mTOR in sun-exposed skin (Figure 3(a)). For AK skin, there was a significant negative correlation between Claudin-1-M or Claudin-1-C and Claudin-1-N, while a strong positive correlation was found between Claudin-1-M and Claudin-1-C. Similarly, Claudin-7-N was negatively correlated with Claudin-1-M. Like in sun-exposed skin, there was no correlation between subcellular TJPs and p-ERK or p-AKT and p-mTOR in the AK group (Figure 3(b)). In the BD group, Claudin-1-C was strongly positively correlated with Claudin-1-M, while Claudin-1-N was negatively correlated with Claudin-1-M and Claudin-1-C, but positively correlated with Claudin-7-N. Meanwhile, Claudin-7-M was positively correlated with Claudin-1-M, Claudin-1-C, and Claudin-7-C, but negatively correlated with Claudin-1-N. Claudin-1-N exhibited a moderate positive correlation with Claudin-7-N. Surprisingly, there were obvious positive correlations among p-ERK, Claudin-1-N, and Claudin-7-N (Figure 3(c)). In SCC samples, there was a strong positive correlation between Claudin-1-C and Claudin-1-M and a strong negative correlation between Claudin-7-N and Claudin-7-M. Significant positive correlations were also found between p-AKT and Claudin-1-C and p-mTOR and Claudin-1-N (Figure 3(d)).

### 3.4. Delocalization of Claudin-1 and Claudin-7 in HaCaT and A431 Cell Lines

**Figure 4:**
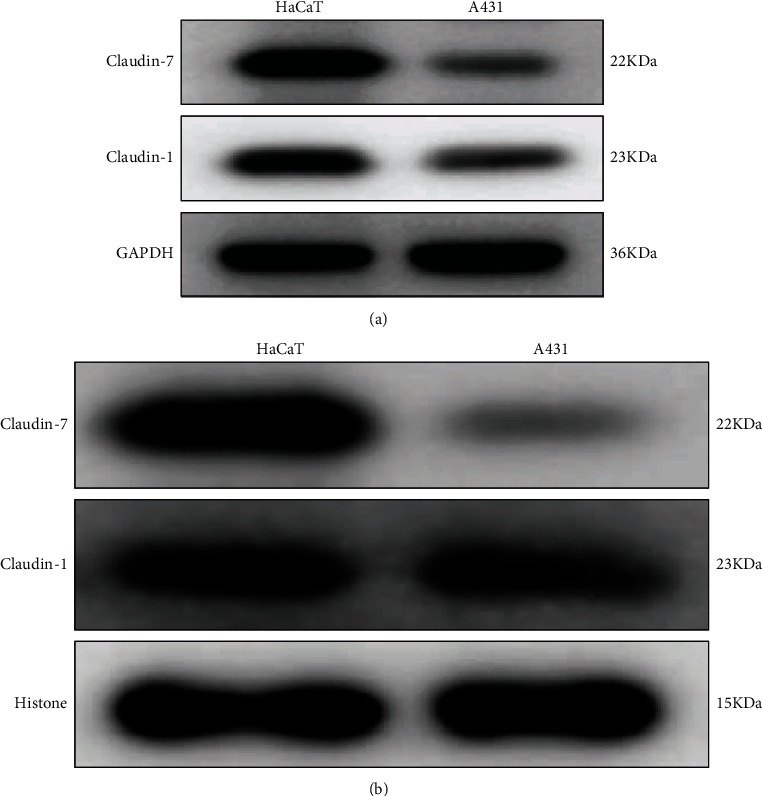
Delocalization of Claudin-1 and Claudin-7 in HaCaT and A431 cells as demonstrated by western blotting. (a) Expression of Claudin-1 and Claudin-7 in the cytoplasm in HaCaT and A431 cells. (b) Expression of Claudin-1 and Claudin-7 in the nucleus in HaCaT and A431 cells.

To further confirm the observation of Claudin-1 and Claudin-7 delocalization in cutaneous SCC and its precancerous lesions, we successively extracted cytoplasmic and nuclear proteins from both HaCaT and A431 cells by western blot analysis. As shown in Figure 4, both Claudin-1 and Claudin-7 were delocalized to the cytoplasm and nucleus of the two types of cells. Inconsistent with markedly reduced Claudin-1 and Claudin-7 delocalization to the cytoplasm (Figure 4(a)), nuclear Claudin-1 was examined with slight reduction but no significant alternation in A431 cells compared with HaCaT cells (Figure 4(b)). However, Claudin-7 remained dramatically nuclear delocalization in A431 cells relative to HaCaT cells (Figure 4(b)). Collectively, the overall reduced Claudin-1 and Claudin-7 delocalization to the cytoplasm and nucleus in A431 cells might indicate their roles in tumorigenesis.

## 4. Discussion

In this study, we determined the subcellular distribution of TJPs in stepwise skin malignancies, such as epithelial dysplasia (AK), CIS (BD), and cutaneous SCC, along with their associated signaling pathway proteins, followed by support of normal HaCaT keratinocytes and cutaneous SCC A431 cells experiment *in vitro*. We found that they participated in tumorigenesis and infiltration of cutaneous SCC.

TJPs were aberrantly expressed in the subcellular skin regions with malignant lesions. Although Claudin-1 and Claudin-7 were found to have intact membranous expression in sun-exposed skin, slight cytoplasmic and strong positive nuclear immunostaining was observed. In both BD and SCC, nuclear Claudin-1 and Claudin7 were significantly low. Although they displayed no or impaired membrane-integrity, their membranous and cytoplasmic immunoexpression remained high. These findings are in contrast to those obtained from three previous reports on Claudin-1 in cutaneous SCC. Heterogeneously expressed Claudin-1 after keratinization was reported in one study [14], while another reported overexpressed Claudin-1 with focal loss of staining in poorly differentiated areas [12], but a third study showed diminished membranous Claudin-1 in AK compared to normal skin and exclusive cytoplasmic Claudin-1 expression in cutaneous SCC [13]. Several factors relating to technology, experimental settings, and patient group selection in this study might have contributed to these discrepant findings. One drawback of this study is the lack of clinical data on cutaneous SCC and precancerous cases. On the other hand, overexpressed, but actually destructive TJPs might lack membrane integrity accompanied by reduced nuclear Claudin-1 and Claudin-7 translocation in cutaneous SCC processes.

The reason for the translocation of Claudin-1 and Claudin-7 from the membrane to the cytoplasm or nucleus is not clearly known in sun-exposed skin, AK, BD, and SCC. A similar result was obtained in melanocytic lesions; Claudin-1 exhibited reduced nuclear localization but high expression in the cytoplasm in both subcutaneous and visceral metastases. However, there was no nuclear localization in lymph node metastases, indicating its metastatic potential [10]. Overexpression of Claudin-1 directly contributed to melanoma cell invasion, while Claudin-1 knockdown inhibited invasion, which further supports the findings in this study [10]. In addition, subcellular localization of Claudin-1 was reported to be deactivated by protein kinase A (PKA), but not protein kinase C (PKC), resulting in Claudin-1 nuclear sequestration, from the nucleus to the cytoplasm, although both PKA and PKC phosphorylation resulted in Claudin-1 cytoplasmic distribution [11]. Surprisingly, despite the existence of rich phosphorylated PKA, reduced nuclear Claudin-1 was inexplicable, and therefore, we hypothesize that a designated Claudin-1 threshold is required before exclusion from the nucleus [11]. Consequently, PKC-rich metastatic melanoma cells possess high nuclear Claudin-1, and PKA phosphorylation leads to the translocation of nuclear Claudin-1 to the cytoplasm [11]. Based on the above studies on melanoma, it can be concluded that delocalization of Claudin-1 and Claudin-7 is linked to carcinogenesis, invasiveness, and metastasis. Therefore, it remains to be determined whether the subcellular localization of Claudin-1 and Claudin-7 contributes to cutaneous SCC.

Contrary to these results, several studies have reported that Claudin-1 translocated from the membrane to the cytoplasm and nucleus, which was also associated with carcinogenesis, invasiveness, metastasis, and apoptosis. Claudin-1 was found to be expressed in the membrane and cytoplasm in normal thyroid tissue, although it was found to be slightly expressed in the nucleus in follicular adenoma and primary follicular thyroid carcinoma. Additionally, it was not expressed in the membrane but was highly expressed in the nucleus in metastatic follicular thyroid carcinoma [8]. Transiently transfected nuclear Claudin-1 augmented migration and invasion, and suppression of Claudin-1 by siRNA inhibited migration and invasion [8]. Claudin-1-transduced MDA-MB 361 breast tumor cell spheroids induced significant apoptosis [9]. Cytoplasmic and nuclear Claudin-1 delocalization was associated with decreased apoptosis in nasopharyngeal carcinoma cell lines [7]. Unlike the subjects selected in this study including hyperplasia, dysplasia, in situ, and invasive carcinoma, most above-mentioned findings were limited to the carcinoma group versus normal group and only presented decreased or increased cytoplasm or nuclear expression and location in the simple background of it. Since carcinogenesis is a multistep, multistage, and multifactor process, it should be analyzed in detail throughout all carcinogenic stages, rather than in isolated cancers or noncancer contexts. Consequently, the higher membranous and cytoplasmic Claudin-1 and Claudin-7 expression in SCC might mean classical membranous structure loss, dysfunctions, and even further tumorigenesis. However, reduced nuclear delocalization would indicate its propensity for carcinogenesis, invasiveness, metastasis, and apoptosis in cutaneous SCC by automatic quantification which was already applied in oral precancerous tissues and oral squamous cell carcinoma [17].

The mechanism behind subcellular localization and alternation of TJPs in cancers remains to be explored. Here, we propose the following hypotheses. Firstly, considerable attention should be given to the nutrient supply hypothesis, in which tumor cells adopt uncontrolled and unlimited access to nutrients due to increased paracellular permeability caused by TJP deficiency [18, 19]. Claudin-1-transduced 3D in breast tumor spheroid culture displayed plasma membrane homing and reduced paracellular flux, which further supports this hypothesis [9]. Interestingly, this study presents a paradoxical situation, where membranous Claudin-1 in BD and Claudin-7 in SCC were highly scored, compared to the classical membrane location, which further indicates that dysregulation of TJ dynamics is linked to carcinogenesis. Additionally, Claudin-1 is a cancer invasion/metastasis suppressor, prognostic predictor, and a potential drug [20]. Accordingly, it was speculated that Claudin-1-transduced cutaneous SCC, although its precancerous cell might show similar membrane homing, paracellular flux inhibition, and increased apoptotic capacity, implying that it could be a therapeutic strategy.

Correlations among subcellular Claudin-1, Claudin-7, p-ERK, p-AKT, and p-mTOR were analyzed at different stages of cutaneous SCC. Subcellular distribution of Claudin-1 and Claudin-7 was correlated with the progress of cutaneous SCC, possibly indicating that altered membrane expression contributes to their mislocalization and potential Claudin-Claudin interaction. These possibilities need to be explored further [21, 22]. Additionally, it was observed that p-ERK correlated with Claudin-1-N and Claudin-7-N in BD, which might explain why p-ERK participates in Claudin-1 and Claudin-7 nuclear delocalization as well as in carcinogenesis. Moreover, p-AKT was positively correlated with Claudin-1-C, while p-mTOR was correlated with Claudin-1-N in cutaneous SCC, which points to their role in carcinogenesis.

## 5. Conclusion

In conclusion, the results presented here indicate that the subcellular localization of Claudin-1 and Claudin-7 and the correlations between them, and with PI-3K/AKT and MEK/ERK signaling pathways, play roles in cutaneous SCC tumorigenesis. The mechanism behind the TJPs delocalization and its associated signaling pathways in cutaneous SCC tumorigenesis, infiltration, and metastases requires further research.

## Figures and Tables

**Table 1:** Staining expression value of tight junction proteins and signaling pathway proteins.

Specimens	Claudin-1		Claudin-7		p-ERK		p-AKT		p-mTOR	
	Mean ± SEM	*n*	Mean ± SEM	*n*	Mean ± SEM	*n*	Mean ± SEM	*n*	Mean ± SEM	*n*
NS										
Mem										
IM scores	3.19 ± 1.77	14	5.81 ± 1.49	7	—	14	—	4	—	11
Cyto										
HP (%)	2.13 ± 2.69		1.63 ± 1.81		16.78 ± 8.33		4.35 ± 0.79		1.54 ± 1.49	
P (%)	11.78 ± 8.10		12.86 ± 6.15		43.50 ± 10.44		26.83 ± 0.31		13.98 ± 7.36	
LP (%)	30.07 ± 7.35		58.30 ± 3.69		37.71 ± 15.87		37.02 ± 1.56		48.81 ± 7.17	
Neg (%)	56.03 ± 15.64		27.36 ± 7.01		2.00 ± 2.43		31.80 ± 0.99		35.67 ± 13.29	
Nuc										
HP (%)	24.99 ± 9.75		18.22 ± 7.39		44.28 ± 9.25		20.73 ± 9.52		21.47 ± 5.31	
P (%)	54.18 ± 5.31		30.91 ± 4.96		43.36 ± 4.70		36.31 ± 3.36		35.02 ± 5.00	
LP (%)	19.20 ± 7.56		33.95 ± 3.87		10.85 ± 4.61		31.17 ± 5.52		33.79 ± 4.00	
Neg (%)	1.63 ± 1.06		16.91 ± 8.44		1.50 ± 0.74		11.79 ± 6.72		9.72 ± 3.82	
AK										
Mem										
IM scores	2.25 ± 1.05	9	2.67 ± 1.12	10	—	10	—	7	—	10
Cyto										
HP (%)	1.83 ± 1.81		0.75 ± 1.00		25.20 ± 15.00		2.53 ± 2.61		7.24 ± 6.42	
P (%)	12.13 ± 7.84		9.64 ± 6.02		49.75 ± 7.72		14.06 ± 4.35		34.85 ± 11.21	
LP (%)	33.11 ± 3.90		41.53 ± 6.96		23.32 ± 10.20		30.15 ± 4.35		44.88 ± 10.80	
Neg (%)	52.93 ± 11.71		47.97 ± 11.97		1.72 ± 1.09		53.26 ± 13.21		13.03 ± 7.49	
Nuc										
HP (%)	31.14 ± 13.10		42.70 ± 10.52		47.20 ± 6.25		38.56 ± 5.45		37.72 ± 10.42	
P (%)	39.28 ± 12.1		41.68 ± 3.80		33.62 ± 4.65		37.67 ± 4.79		31.31 ± 4.05	
LP (%)	22.77 ± 12.35		13.82 ± 7.89		16.54 ± 4.23		20.86 ± 3.74		22.62 ± 5.49	
Neg (%)	6.83 ± 12.2		1.79 ± 1.85		3.66 ± 1.72		3.97 ± 2.68		8.35 ± 5.68	
BD										
Mem										
IM scores	5.19 ± 2.57	14	4.75 ± 3.23	14	—	13	—	15	—	14
Cyto										
HP (%)	5.79 ± 8.40		0.97 ± 1.13		17.83 ± 11.54		3.03 ± 2.52		3.09 ± 4.06	
P (%)	19.16 ± 10.35		9.83 ± 8.52		41.46 ± 8.50		19.73 ± 12.91		25.60 ± 12.36	
LP (%)	32.16 ± 4.20		41.79 ± 14.18		36.30 ± 14.32		30.97 ± 9.17		51.33 ± 6.88	
Neg (%)	42.83 ± 15.93		47.54 ± 19.35		4.42 ± 3.01		46.27 ± 21.93		19.98 ± 13.97	
Nuc										
HP (%)	25.37 ± 11.70		31.07 ± 11.63		38.77 ± 18.44		26.33 ± 7.72		24.72 ± 10.85	
P (%)	42.98 ± 7.11		47.35 ± 11.99		34.23 ± 7.67		42.83 ± 4.47		30.60 ± 3.56	
LP (%)	26.15 ± 11.01		18.83 ± 8.00		21.41 ± 12.67		25.75 ± 7.27		30.52 ± 6.15	
Neg (%)	5.53 ± 5.63		2.46 ± 1.68		5.60 ± 5.61		5.09 ± 3.73		14.05 ± 7.17	
SCC										
Mem										
IM scores	3.36 ± 1.67	11	11.46 ± 3.77	8	—	10	—	11	—	9
Cyto										
HP (%)	1.92 ± 2.22		1.34 ± 2.06		7.28 ± 4.68		3.90 ± 3.67		2.62 ± 2.80	
P (%)	11.51 ± 8.91		16.01 ± 14.35		34.25 ± 12.91		14.94 ± 6.84		27.12 ± 11.11	
LP (%)	33.11 ± 3.90		56.48 ± 4.89		49.56 ± 8.95		29.41 ± 5.33		52.87 ± 5.58	
Neg (%)	58.83 ± 18.82		26.23 ± 13.54		8.90 ± 9.60		51.75 ± 12.73		17.39 ± 8.95	
Nuc										
HP (%)	17.69 ± 8.64		11.31 ± 12.40		24.10 ± 8.20		22.65 ± 6.97		16.69 ± 12.22	
P (%)	36.58 ± 10.1		20.54 ± 5.57		40.03 ± 6.26		42.96 ± 7.17		24.47 ± 7.22	
LP (%)	35.14 ± 9.36		33.05 ± 6.39		29.47 ± 8.26		29.79 ± 8.62		35.00 ± 6.19	
Neg (%)	10.3 ± 6.26		35.10 ± 12.59		6.39 ± 3.08		4.59 ± 2.41		23.84 ± 13.10	

NS: sun-exposed skin; AK: actinic keratosis; BD: Bowen's disease; SCC: squamous cell carcinoma; Mem: membrane; Cyto: cytoplasm; Nuc: nucleus; HP: high positive; P: positive; LP: low positive; Neg: negative.

## Data Availability

The data used to support the findings of this study are included within the article. Further inquiries can be directed to the corresponding author.
